# PU.1: the conductor of Alzheimer’s symphony

**DOI:** 10.1186/s13024-026-00937-1

**Published:** 2026-03-13

**Authors:** Chunli Sun, Qianjin Liu

**Affiliations:** Jiangsu Key Laboratory of Brain Disease Bioinformation, Research Center for Biochemistry and Molecular Biology, Xuzhou Medical University, Xuzhou, 221004 China

**Keywords:** Alzheimer’s disease, PU.1, Microglia

## Abstract

A recent study by Ayata et al. (2025) reveals that the transcription factor PU.1 acts as a molecular switch that determines whether microglia protect against or worsen Alzheimer’s disease pathology, with lower PU.1 levels promoting beneficial microglial states that reduce amyloid burden and neuroinflammation.

Microglia, the brain’s resident immune cells, play multifaceted roles in health and disease. In Alzheimer’s disease (AD), they are among the responders to amyloid deposition, migrating toward plaques where they can either promote clearance or exacerbate inflammation and neurodegeneration [1]. Understanding what determines these opposing phenotypes has been a long-standing challenge in neuroimmunology. A recent study published in Nature [2], Pinar Ayata, Jessica M. Crowley and colleagues identifies the transcription factor PU.1 (Spi1) as a master regulator that fine-tunes microglial identity and function, revealing how subtle modulation of its activity can pivot microglia from a pathogenic to a protective state.

PU.1 is a non-canonical pioneer transcription factor that orchestrates the differentiation programs of both myeloid and lymphoid lineages. Beyond hematopoiesis, it serves as a pivotal transcriptional determinant governing microglial specification and maturation [3]. In recent years, accumulating genetic and functional evidence has implicated altered PU.1 abundance in microglia as a contributor to AD susceptibility. Human genetic data have pinpointed SPI1, the gene encoding PU.1, as a key GWAS risk locus for AD, with multiple lines of evidence suggesting it may be the key gene within this locus or at least one of the genes responsible for the observed association with AD. Intriguingly, individuals exhibiting lower SPI1 expression in myeloid cells tend to experience a delayed onset and attenuated progression of the disease, suggesting that reduced PU.1 activity may exert a protective influence [3]. However, functional studies have yielded seemingly conflicting results: some reports indicate that reduced PU.1 expression confers neuroprotection, whereas others suggest that lower PU.1 levels are associated with increased AD risk [4–8]. These contrasting findings highlight the intriguing and context-dependent role of PU.1 in shaping disease progression, making it one of the most captivating transcriptional regulators in Alzheimer’s research.

In this article, through an elegant combination of single-cell transcriptomics, chromatin accessibility profiling, and in vivo manipulation, Pinar Ayata, Jessica M. Crowley and colleagues uncover a previously unrecognized subset of PU.1^low^ plaque-associated microglia (PAMs) enriched around amyloid plaques. Unlike conventional homeostatic or disease-associated microglia, these PU.1^low^ cells express a suite of genes characteristic of lymphoid immune regulation—including CD28, PD-1, and CTLA-2A—and display resistance to CSF1R inhibition, suggesting an alternative survival program. Mechanistically, SYK–PLCγ2 signaling downstream of receptors such as TREM2 mediates local PU.1 downregulation, which in turn reshapes chromatin accessibility and reprograms transcription toward a regulatory, lymphoid-like state.

Functionally, reducing PU.1 expression in microglia produces a marked shift in disease trajectory. In 5xFAD mice, microglia-specific PU.1 knockdown enhances amyloid plaque morphology, lowers total Aβ burden, dampens neuroinflammatory gene expression, and limits the spread of phosphorylated tau. These protective effects coincide with the emergence of CD28⁺ PU.1^low^ microglia, which exhibit immunosuppressive transcriptional signatures and support neuronal survival. Conversely, elevating PU.1 levels amplifies inflammatory activation and worsens pathology, highlighting the dosage-sensitive role of this transcriptional regulator (Fig. 1).

**Fig. 1:**
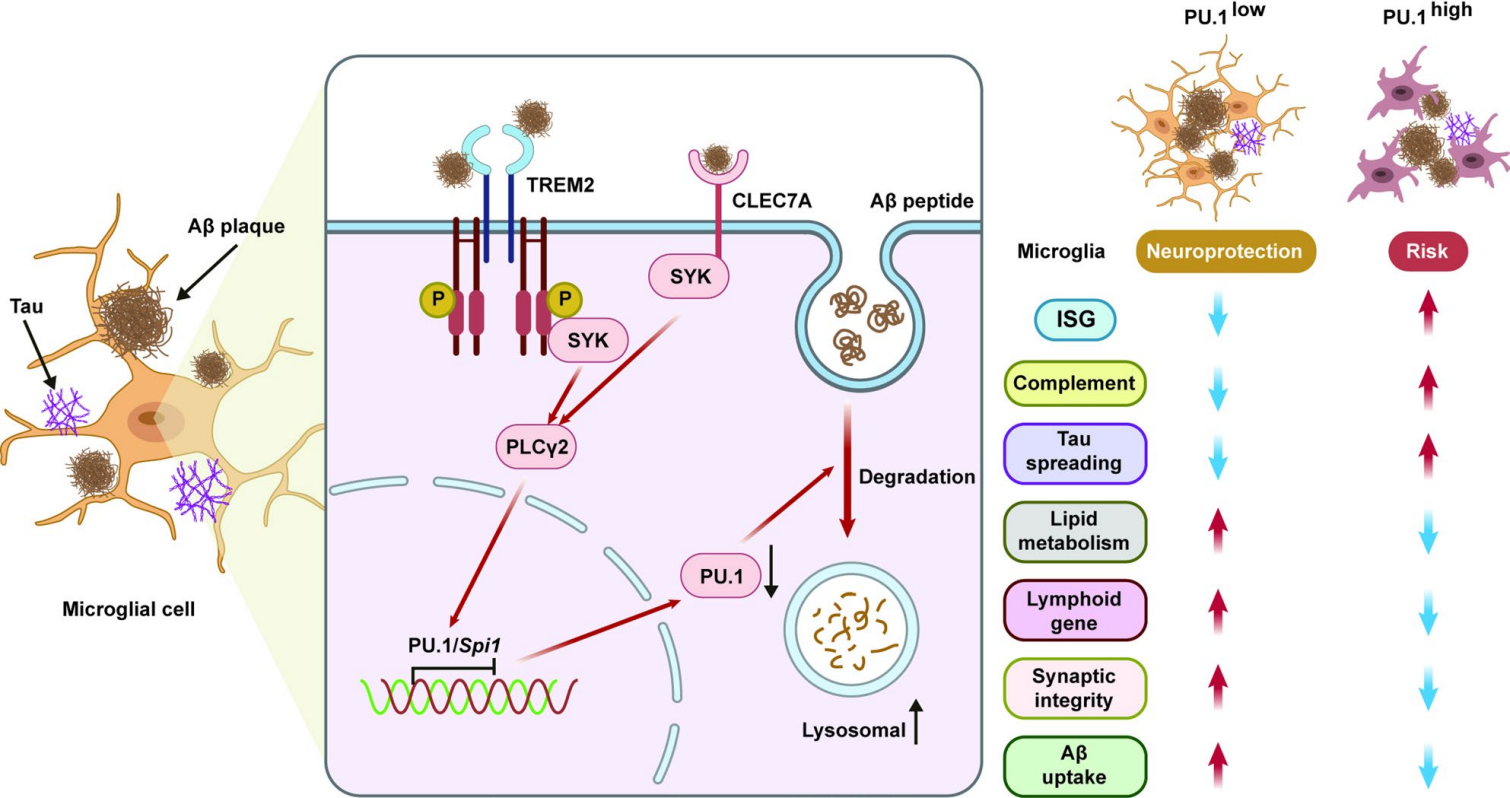
PU.1 modulation orchestrates microglial identity in Alzheimer’s disease. Amyloid pathology activates SYK–PLCγ2 signaling, leading to local PU.1 downregulation in plaque-associated microglia. PU.1^low^ microglia adopt a lymphoid-like regulatory phenotype characterized by CD28, PD-1, and CTLA-2A expression. Functionally, these PU.1 low microglia compact amyloid plaques, reduce Aβ load, and limit tau propagation, thus restraining neuroinflammation and neurodegeneration

This discovery adds an important dimension to our understanding of microglial plasticity in neurodegeneration. PU.1, traditionally viewed as a lineage-defining factor for myeloid cells, now emerges as a molecular rheostat capable of rewiring microglial identity within the diseased brain. The finding of the PU.1 function in microglia has been solved to a certain extent the opposing hypotheses on the roles of PU.1 in microglial activity during AD disease programming. Notably, genetic studies in humans echo the founding of this article: allelic variants associated with reduced PU.1 expression correlate with delayed AD onset, reinforcing the translational relevance of this pathway. Despite its promising translational potential, PU.1 is not confined to the central nervous system; Since PU.1 also plays a significant role in peripheral immune systems, direct manipulation of its expression or activity may lead to peripheral immune side effects. Therefore, future strategies would need to consider more precise targeting methods.

Beyond its mechanistic insight, this work carries broad therapeutic implications. Targeting PU.1 dosage or mimicking the PU.1^low^ transcriptional program could represent a strategy to recalibrate microglial function without compromising their essential homeostatic roles. Small-molecule inhibitors, epigenetic modulators, or RNA-based interventions may offer routes to achieve this reprogramming. Given the difficulty in AD staging, PU.1-targeted therapies may require consideration of the disease’s stage, as the effects of PU.1 activity could vary depending on the progression of the disease. Thus, more personalized approaches would be needed. Moreover, the identification of CD28 as a functional marker of the protective microglial state invites exploration into whether classical co-stimulatory signaling mechanisms—long studied in T cells—operate analogously in the brain’s innate immune cells. In the periphery, CD28-null T cells are linked to chronic inflammation in diseases such as rheumatoid arthritis and multiple sclerosis. In parallel, CD28^+^ microglia in AD help reduce neuroinflammation and promote neuronal survival, suggesting a conserved immunoregulatory role analogous to that of CD28^+^ T cells. This parallel implies that CD28^+^ microglia may represent a promising therapeutic target, similar to strategies targeting CD28 in peripheral immune disorders. Notably, species-specific differences in CD28 [9], including a non-conserved amino acid variant that leads to divergent signaling, suggest that the protective functions of CD28^+^ microglia observed in Ayata et al.’s mouse model may not directly translate to humans. Therefore, cautious interpretation and validation in human microglial systems are required.

While these findings are promising, further exploration is needed to assess the robustness of PU.1 manipulation compared to other immune system interventions. More rigorous studies are required to determine whether PU.1 could serve as a viable therapeutic target for AD or if alternative immune strategies may prove more effective. Additionally, this study advances understanding of the dual role of immune engagement in AD and aging, where microglial activity can shift between protective and detrimental states, depending on the regulatory signals involved.

Several open questions remain: How stable is the PU.1^low^ state, and what environmental signals maintain it? Does PU.1 interact with other Alzheimer’s-related regulators such as Nrf2, or NF-κB, integrating redox and inflammatory cues into a unified transcriptional response? Given that the PI3K/AKT/mTOR signaling pathway has been reported to play an important role in Spi1-mediated regulation of microglial and macrophage inflammatory responses during intracerebral hemorrhage [10], might this pathway similarly contribute to the regulation of glial inflammation by PU.1^low^ microglia in AD? Finally, can transient induction of PU.1^low^ microglia confer durable neuroprotection, or is continuous tuning required to sustain resilience?

Together, these findings reveal a nuanced transcriptional strategy by which the brain’s immune cells adapt to neurodegenerative stress. By fine-tuning, rather than silencing, PU.1 activity, microglia orchestrate a protective symphony—one that balances immune vigilance with restraint, and may ultimately hold the key to tempering inflammation in Alzheimer’s disease.

## Data Availability

Not applicable.

## Abbreviations

AD: Alzheimer’s disease
PAMs: Plaque-associated microglia
